# Development and validation of machine-learning algorithms predicting retention, overdoses, and all-cause mortality among US military veterans treated with buprenorphine for opioid use disorder

**DOI:** 10.1080/10550887.2024.2363035

**Published:** 2024-06-30

**Authors:** Corey J. Hayes, Nahiyan Bin Noor, Rebecca A. Raciborski, Bradley C. Martin, Adam J. Gordon, Katherine J. Hoggatt, Teresa Hudson, Michael A. Cucciare

**Affiliations:** a Department of Biomedical Informatics, College of Medicine, University of Arkansas for Medical Sciences, Little Rock, AR, USA; b Institute for Digital Health and Innovation, College of Medicine, University of Arkansas for Medical Sciences, Little Rock, AR, USA; c Center for Mental Healthcare and Outcomes Research, Central Arkansas Veterans Healthcare System, North Little Rock, AR, USA; d Behavioral Health Quality Enhancement Research Initiative, Central Arkansas Veterans Healthcare System, North Little Rock, AR, USA; e Evidence, Policy, and Implementation Center, Central Arkansas Veterans Healthcare System, North Little Rock, AR, USA; f Division of Pharmaceutical Evaluation and Policy, College of Pharmacy, University of Arkansas for Medical Sciences, Little Rock, AR, USA; g Program for Addiction Research, Clinical Care, Knowledge, and Advocacy (PARCKA), Division of Epidemiology, Department of Medicine, School of Medicine, University of Utah, Salt Lake City, UT, USA; h Informatics, Decision-Enhancement and Analytic Sciences (IDEAS) Center, VA Salt Lake City Healthcare System, Salt Lake City, UT, USA; i San Francisco VA Medical Center, San Francisco, CA, USA; j Department of Medicine, University of California, San Francisco, San Francisco, CA, USA; k Center for Health Services Research, Department of Psychiatry, College of Medicine, University of Arkansas for Medical Sciences, Little Rock, AR, USA; l Department of Emergency Medicine, College of Medicine, University of Arkansas for Medical Sciences, Little Rock, AR, USA; m Veterans Affairs South Central Mental Illness Research, Education and Clinical Center, Central Arkansas Veterans Healthcare System, North Little Rock, AR, USA

**Keywords:** Veterans, buprenorphine, opioid use disorder, predictive modeling, machine-learning algorithms

## Abstract

**Background:**

Buprenorphine for opioid use disorder (B-MOUD) is essential to improving patient outcomes; however, retention is essential.

**Objective:**

To develop and validate machine-learning algorithms predicting retention, overdoses, and all-cause mortality among US military veterans initiating B-MOUD.

**Methods:**

Veterans initiating B-MOUD from fiscal years 2006–2020 were identified. Veterans’ B-MOUD episodes were randomly divided into training (80%;*n* = 45,238) and testing samples (20%;*n* = 11,309). Candidate algorithms [multiple logistic regression, least absolute shrinkage and selection operator regression, random forest (RF), gradient boosting machine (GBM), and deep neural network (DNN)] were used to build and validate classification models to predict six binary outcomes: 1) B-MOUD retention, 2) any overdose, 3) opioid-related overdose, 4) overdose death, 5) opioid overdose death, and 6) all-cause mortality. Model performance was assessed using standard classification statistics [e.g., area under the receiver operating characteristic curve (AUC-ROC)].

**Results:**

Episodes in the training sample were 93.0% male, 78.0% White, 72.3% unemployed, and 48.3% had a concurrent drug use disorder. The GBM model slightly outperformed others in predicting B-MOUD retention (AUC-ROC = 0.72). RF models outperformed others in predicting any overdose (AUC-ROC = 0.77) and opioid overdose (AUC-ROC = 0.77). RF and GBM outperformed other models for overdose death (AUC-ROC = 0.74 for both), and RF and DNN outperformed other models for opioid overdose death (RF AUC-ROC = 0.79; DNN AUC-ROC = 0.78). RF and GBM also outperformed other models for all-cause mortality (AUC-ROC = 0.76 for both). No single predictor accounted for >3% of the model’s variance.

**Conclusions:**

Machine-learning algorithms can accurately predict OUD-related outcomes with moderate predictive performance; however, prediction of these outcomes is driven by many characteristics.

## Introduction

In the US, the opioid crisis is one of the most costly public health emergencies, both in terms of lives lost and health expenditures.^1–3^ Since 2010, deaths involving opioids have risen by over 300%, increasing from 21,089 in 2010 to 80,411 overdose deaths in 2021.^4^ The opioid crisis has also significantly affected the U.S. economy; a new federal report estimated healthcare costs related to the opioid epidemic were $1.5 trillion in 2020 alone, a 52% increase from 2019.^5^ Following the national trend, the number of US military veterans diagnosed with OUD has nearly tripled over the last 15 years.^6,7^

Medication treatment for opioid use disorder (MOUD; formulations of buprenorphine, methadone, and naltrexone) is the standard of care for treatment of OUD because it prevents overdoses and reduces drug use.^8–10^ However, retention on MOUD is essential to attaining these outcomes.^11,12^ In recent years, the Veterans Health Administration (VHA) has improved veterans’ access to MOUD, particularly for buprenorphine, since it can easily be attained from pharmacies and health care clinics including primary care.^13^ Due to VHA efforts,^7,14^ the proportion of Veterans receiving any MOUD increased from 34.6% to 48.9% from 2015 to 2020, and buprenorphine accounts for over 80% of MOUD in VHA.^15^ Despite these gains in initiating treatment with buprenorphine, positive reductions in overdose are only achieved with adequate MOUD retention.^11,12^ Historically, the median duration of treatment with buprenorphine is 157 days.^16^ Negative treatment experiences and not obtaining additional needed care services (e.g., counseling to address mental health comorbidities) are important risk factors for early (i.e.,<180 days) MOUD discontinuation, a minimum standard set by the National Quality Forum.^17,18^ Consequently, providers’ ability to identify veterans in need of additional support while on MOUD holds promise for increasing MOUD retention.

Predictive modeling is a real-time method to estimate a person’s probability of experiencing an outcome of interest at some point in the future. VHA currently uses clinical decision support tools based on predictive models to identify veterans at high risk for opioid overdose and suicide and to provide those veterans with additional care to improve their outcomes.^19,20^ Specifically, VHA developed Recovery Engagement and Coordination for Health-Veterans Enhanced Treatment (REACH VET) and the Stratification Tool for Opioid Risk Mitigation (STORM) dashboards, which are based on predictive models. These dashboards are used daily in VHA clinical care. Theoretically, similar clinical decision support tools could be used to reduce MOUD attrition and fatal and non-fatal overdoses among veterans with OUD. This tool could identify veterans at risk for MOUD discontinuation or overdose who may benefit from additional support including peer support, outpatient counseling, and pharmacotherapy. This tool could also identify potentially modifiable risk factors, including buprenorphine dose, polysubstance use, mental health comorbidities that could be treatment targets to improve veterans’ treatment retention and decrease their risk for overdose. The primary goal of this study was to develop and validate machine-learning algorithms to predict retention of buprenorphine treatment for veterans with OUD (B-MOUD), their fatal and non-fatal overdoses, and all-cause mortality in the year following a B-MOUD treatment episode.

## Methods

### Data sources

We used inpatient, outpatient, demographic, and outpatient pharmacy files from the VHA Corporate Data Warehouse (CDW) from fiscal years 2006 through 2020 (October 1, 2005 – September 30, 2020). Data are stored in the CDW relationally based on the original contributing source [e.g., electronic health record (EHR), eligibility file, vital signs data]; study personnel linked data from the extracts of the inpatient, outpatient, demographic, pharmacy, to construct an analytic file. Linking of datasets within the CDW (e.g., inpatient, outpatient, eligibility files) were done using scrambled social security numbers. EHR data were also linked with the U.S. Centers for Disease Control and Prevention/Agency for Toxic Substances and Disease Registry Social Vulnerability Index (CDC/ATSDR SVI) themes using the veteran’s zip code of residence. Specifically, we used the SASHELP. zipcode file to link the veteran’s zip code to the veteran’s county and then linked to the county version of the CDC/ATSDR SVI. The constructs of the SVI are continuous variables of four geographically defined vulnerability domains: (1) socioeconomic status (Theme 1), (2) household composition and disability (Theme 2), (3) minority status and language (Theme 3), (4) housing and transportation (Theme 4).^21^

### Study design and subjects

This study was approved by the Central Arkansas Veterans Healthcare System Institutional Review Board (Little Rock, AR; Date of Approval: 10/5/2020; Approval Number: 1578556). This study followed reporting guidelines from the Standards for Reporting of Diagnostic Accuracy (STARD) and the Transparent Reporting of Multivariable Prediction Model for Individual Prognosis or Diagnosis (TRIPOD).^22,23^

We identified veterans who received at least one OUD diagnosis, were at least 18 years of age, and had initiated B-MOUD after receiving an OUD diagnosis between fiscal years 2006–2020. OUD diagnoses were operationalized to include the specific diagnoses below as used by Lagisetty et al., 2021.^24^ The study period crosses over the US implementation of the International Classification of Diseases, 10th Revision, Clinical Modification (ICD-10-CM) on Oct 1, 2015; therefore, we used ICD-9-CM and ICD-10-CM codes to operationalize OUD. Eligible codes for defining OUD in the primary analysis are shown in eTable 1. Initiation of B-MOUD was defined as a prescription fill from the VHA outpatient pharmacy or from barcode administration (e.g., inpatient administration) as defined similarly to a previous MOUD algorithm.^25^ B-MOUD episodes started on the date of a B-MOUD prescription fill. B-MOUD retention was defined as having at least 180 days of B-MOUD coverage after the start of the B-MOUD episode without a gap of 30 days or more in coverage.^26^ Veterans could have multiple B-MOUD episodes throughout the study timeframe, and all unique episodes were considered in the analysis.^16^ An illustration of multiple episodes can be found in eFigure 1.

### Exclusion criteria

We implemented 4 exclusion criteria based on the CDW records: (1) receipt of B-MOUD from a non-VA source or as a part of a clinical trial, (2) receipt of B-MOUD before an OUD diagnosis was recorded in CDW, (3) enrollment date after B-MOUD initiation, and (4) missing key data elements for VHA priority status, race/ethnicity, CDC/ATSDR SVI themes, marital or employment status, facility type of B-MOUD initiation, VA disability or pension status, smoking status, and Rural-Urban Commuting Area (RUCA) codes.

### Study outcomes

We built and validated prediction models for one primary outcome (retention on B-MOUD) and five secondary outcomes (binary measures for fatal and non-fatal overdose, fatal and non-fatal opioid overdose, overdose death, opioid overdose death, and all-cause mortality). Our primary outcome, retention on B-MOUD, was defined as a binary measure of continuous coverage with B-MOUD for at least 180 days after treatment initiation without a 30-day gap in treatment.^27^ Our secondary outcomes were measured over the 365 days after the start of the B-MOUD episode. Fatal overdoses and all-cause mortality were defined using the VHA/DoD Mortality Data Repository.^28^ A death was counted as an opioid overdose death or overdose death if it was recorded on the death certificate as resulting from an opioid overdose or overdose as represented by ICD-9-CM and ICD-10-CM codes.^29^ Similarly, non-fatal overdoses were derived from VHA inpatient or outpatient visits and healthcare visits to non-VHA facilities yet paid for by VHA as represented by ICD-9-CM and ICD-10-CM codes.^29^ Of note, fatal and non-fatal overdoses is inclusive of overdose death, and fatal and non-fatal opioid overdoses is inclusive of opioid overdose death.

### Candidate predictors

We identified 114 candidate predictors based on prior evidence for their association with either MOUD retention or opioid overdose.^3,30–39^ Patient-, provider-, and facility-level candidate predictors were measured over the 365 days prior to start of the B-MOUD episode. Patient-level candidate predictors included: (1) sociodemographic factors, (2) MOUD profile characteristics, (3) health care service utilization, (4) disease comorbidity factors, (5) opioid-specific historical factors, (6) non-opioid prescription medication utilization factors, (7) social risk factors, (8) provider-level factors, and (9) facility-level factors. Sociodemographic factors included age, sex, ethnicity, race, employment status, marital status, and VHA priority status (a proxy for a veteran’s overall health need).^40^ B-MOUD profile characteristics included dose of B-MOUD at treatment initiation, treatment setting in which the veteran initiated B-MOUD, days’ supply of B-MOUD received on the first day of the B-MOUD episode, and characteristics about prior B-MOUD episodes. Health care service utilization included the number of emergency room visits, receipt of psychotherapy, or having a general inpatient or psychiatric inpatient admission. Disease comorbidity factors included the Elixhauser comorbidity count^41^ and concurrent diagnoses of tobacco use disorder, alcohol use disorder, other drug use disorder, major depression, psychotic disorder, post-traumatic stress disorder, anxiety disorders, bipolar disorder, hepatitis C, and chronic pain. The Elixhauser Comorbidity Index, of which we just use the count of comorbidities in the Index, is a method of categorizing comorbidities using ICD codes to measure overall severity of comorbidities. The Index has been used to predict mortality.^42,43^ Opioid-specific historical factors included opioid overdose in the 365 days prior to the start of the B-MOUD episode and receipt of prescription opioids in the 30 days prior to the start of the B-MOUD episode. Non-opioid prescription medication utilization factors included receipt of sedatives, benzodiazepines, or antidepressants in the 30 days prior to the B-MOUD episode initiation date. Social risk factors included rural residence,^44^ justice-involvement, unhoused status,^45^ and SVI themes.^21^ Provider-level candidate predictors included the provider’s specialty (e.g., Addiction Medicine, Emergency Medicine), provider’s credential (e.g., MD, PA, PharmD), number of MOUD prescriptions the provider wrote in the year prior to the episode initiation date, and the percent of patients the provider retained on MOUD in the prior year. Facility-level candidate predictors included at which VHA facility the veteran initiated the B-MOUD episode, the percentage of patients at the given facility being treated with MOUD in the year prior, and whether the facility has an inpatient detoxification or opioid treatment program. eTables 2 and 3 contain the full lists all candidate predictors.

### Machine learning Methods and evaluation of predictive performance

To build and validate models to predict (classification) our primary and secondary outcomes, we divided B-MOUD episodes randomly into training (80%) and testing (20%) datasets. We used the training dataset to develop the models (e.g., hyperparameter tuning and model selection) and the hold-out testing dataset to determine the performance of our selected models. We used five candidate machine-learning algorithms: (1) Multiple Logistic Regression (MLR), (2) Least Absolute Shrinkage and Selection Operator-Type Regression (LASSO) (3) Random Forest (RF), (4) Gradient Boosting Machine (GBM), and (5) Deep Neural Network (DNN). These five techniques were chosen because prior research has repeatedly demonstrated that these algorithms produce accurate prediction outcomes in a variety of applications.^46–48^ Details on each of the models can be found in the Appendix.

We used, with each of the model types, the grid search (GridSearchCV package in Python) technique for hyperparameter turning.^49^ Grid search methodically assesses various hyperparameters, measuring model performance via cross-validation, to identify the optimal configuration. Additional details on the hyperparameter tuning process can be found in the Appendix.

We also employed a comprehensive approach to address the imbalance in the datasets, primarily with the secondary outcomes, leveraging three distinct dataset balancing techniques to ensure robustness and reliability of the findings. These techniques are (1) Synthetic Minority Over-Sampling Technique (SMOTE), (2) Under sampling, and (3) Random Over-Sampling Examples (ROSE). Addtional details of these techniques can be found in the Appendix.

A strong evaluative framework using different evaluation metrics was developed to meticulously evaluate model performance among the testing dataset. Since we are focused on classification, we used the area under the receiver operating characteristic curve (AUC-ROC), area under the precision-recall curve (AUC-PR) sensitivity (also known as recall), specificity, positive predictive value (PPV; also known as precision), negative predictive value (NPV), and F1 score to assess the predictive performance of the models. These classification statistics were chosen because they are commonly used in determining clinical utility.^50,51^ The AUC-ROC is a graphical plot of sensitivity and 1-specificity on a continuous scale. An AUC-ROC of 0.5 means prediction is no better than chance since, for a binary outcome, chance provides an accurate prediction 50% of the time. Therefore, an AUC-ROC of 1 is considered perfect prediction. While the AUC-ROC summarizes the trade offs between the true positive and false positive rates, the precision-recall curve summarizes the trade offs between the true positive rate and the PPV. For calculation of other static measures (i.e., sensitivity, specificity, PPV, NPV, and F1 score), we selected a classification threshold to maximize the Youden index.^52^ The Youden Index is a summary measure of accuracy of a test or model which maximizes sensitivity and specificity giving equal weight to each and ranges from 0 to 1.^53^ We also highlight the top predictors with a cumulative importance greater than 80%, a common threshold in machine-learning.^54^ A cumulative importance greater than 80% means the top features up to that point account for 80% of the predictive power or variation explained by the model. We used a combination of one-hot encoding and label encoding to transform the candidate predictors for modeling.^55^ One-hot encoding was used for categorical variables with no ordinal relationship creating a new binary column (0 or 1) for each category level. Label encoding was used for categorical variables with an ordinal relationship between levels; each level was assigned an integer value.

### Statistical analysis

We determined the mean and standard deviation for all continuous candidate predictors and proportions for categorical candidate predictors. We used SAS v8.3 and SQL for data manipulation and Python 3.11.5 for analyses.

### Sensitivity analyses

We conducted two sensitivity analyses. First, we used principal component analysis to condense the candidate predictors down to a given number of components (*n* = 15, 30, and 50).^56^ Second, we limited the predictors to only those with a cumulative importance greater than 80%, following previous methodologies, to test the impact this limitation may have on the predictive performance metrics as a more parsimonious number of predictors can be helpful in clinical implementation.^54^

## Results

### Sample characteristics

A total of 56,547 B-MOUD episodes (from 34,032 Veterans) met study eligibility (eFigure 2). B-MOUD episodes in the training cohort (45,237; 80.0%) and in the testing cohort (11,310; 20.0%) were similar in candidate predictor characteristics and outcomes. Of all episodes in the training cohort, 93.0% were male, 78.0% were White, 19.4% were employed, 72.3% were unemployed, 8.4% were retired and 48.3% had a concurrent drug use disorder (Table 1). For outcomes, 37.6% of episodes were retained on B-MOUD for 180 days, 9.9% experienced a fatal or non-fatal overdose, 4.9% experienced a fatal or non-fatal opioid overdose, 1.3% died of an overdose, 1.1% died of an opioid overdose, and 3.2% died from any cause in the 365 days after B-MOUD initiation for the given episode. The testing cohort had similar characteristics. The counts of each outcome (primary and secondary outcomes) before balancing are shown in eFigure 3.

### Predictive performance: primary outcome of B-MOUD retention

**Figure 1.**
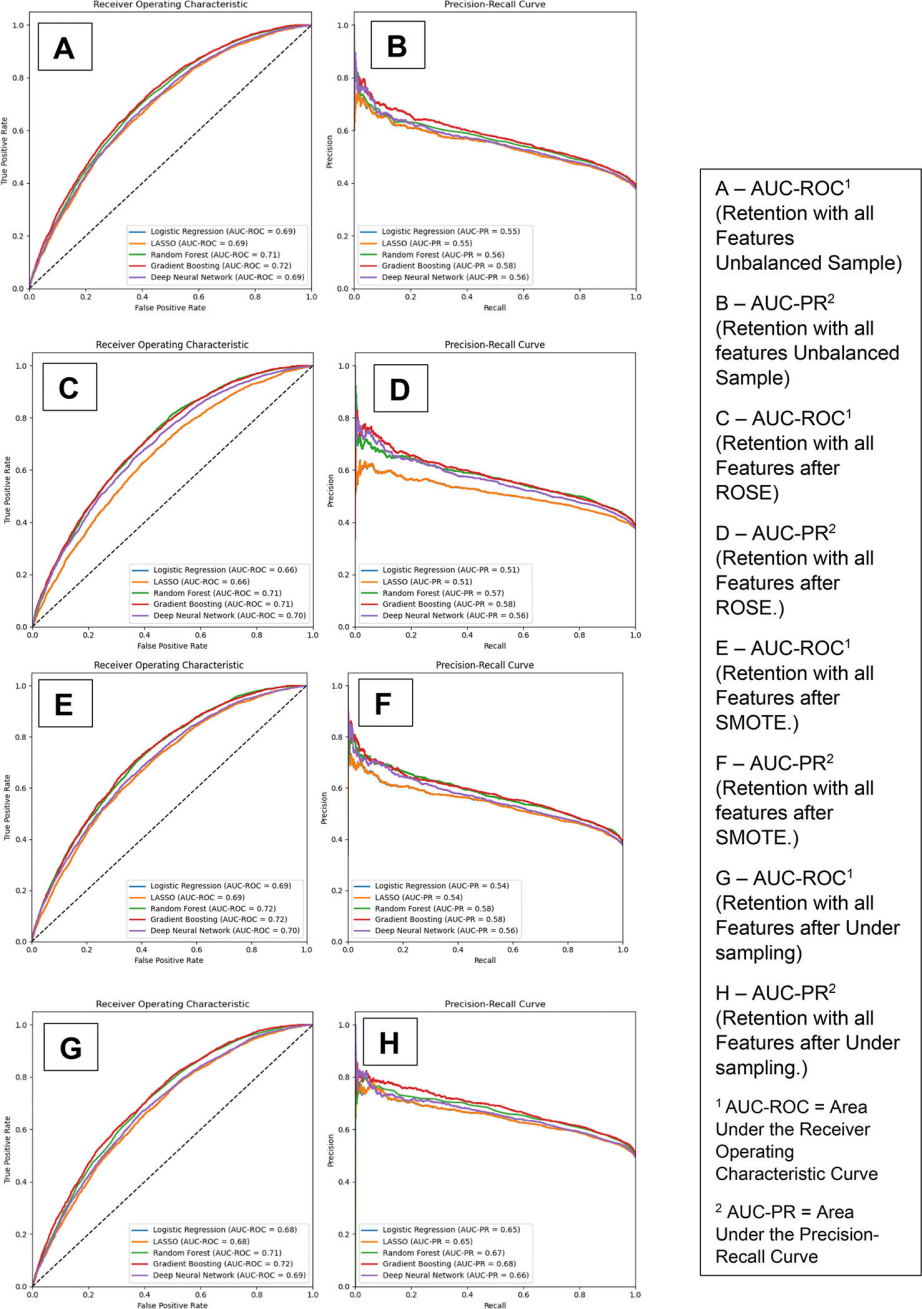
Area under the receiver operating curve and precision-recall curve for B-moud retention among the unbalanced and balanced with smote, rose, and under sampling techniques.

The AUC-ROC and precision-recall curves for our 5 models for B-MOUD retention in the testing sample are displayed in Figure 1. Panels A and B provide the curves for the models with all candidate predictors without a balancing technique. Panels C and D, E and F, and G and H provide the results using the ROSE, SMOTE, and under-sampling balancing techniques respectively. Across techniques, GBM and RF provided the best results. The unbalanced and under-sampling samples had the same performance (GBM AUC-ROC: 0.72, RF AUC-ROC: 0.71). ROSE balancing only slightly improved predictive performance (GBM AUC-ROC: 0.72, RF AUC-ROC: 0.72) while SMOTE balancing had similar performance as the unbalanced and under-sampling (GBM AUC-ROC: 0.71, RF AUC-ROC: 0.71).

eTable 3 provides the optimized sensitivity and specificity based on the Youden index. GBM, among the unbalanced sample, had a sensitivity of 78%, specificity of 54%, PPV of 53%, and NPV of 69%. RF had a sensitivity of 75%, specificity of 55%, PPV of 52%, and NPV of 68% and both LR and LASSO had a sensitivity of 72%, specificity of 55%, PPV of 49%, and NPV of 72%.

**Figure 2.**
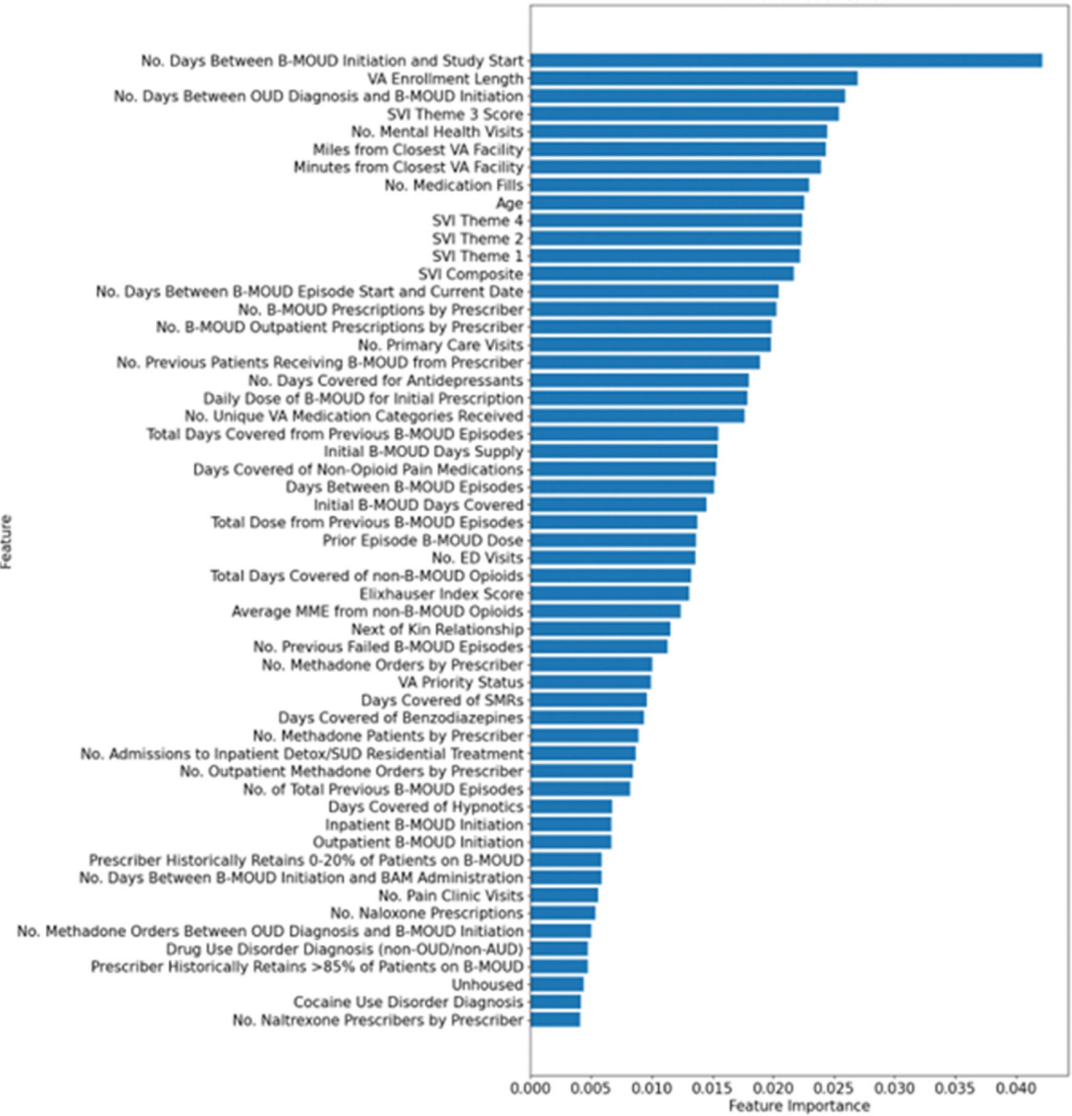
Predictors of most importance for B-moud retention from the random forest model among the unbalanced sample (*N* = 55).

Figure 2 shows the most important predictors (*n* = 55; cut point for cumulative performance of >80%–see Methods) for B-MOUD retention. Noteably, no single predictor contributed more than 4% to the model’s prediction for retention. The five most influential predictors were number of days between B-MOUD initiation and study start (October 1, 2005), length of VA enrollment prior to initiation of B-MOUD episode, number of days between OUD diagnosis and B-MOUD initiation, SVI Theme 3 score (minority status and language), and number of mental health visits prior to B-MOUD initiation.

eFigures 4–6 show the most important predictors (*n* = 61; *n* = 55; *n* = 52 cut point for cumulative performance of >80%) for B-MOUD discontinuation after applying SMOTE, ROSE and under-sampling respectively. The most influential predictors were largely the same as in the original model based on unbalance data.

### Predictive performance: Secondary outcomes

**Figure 3.**
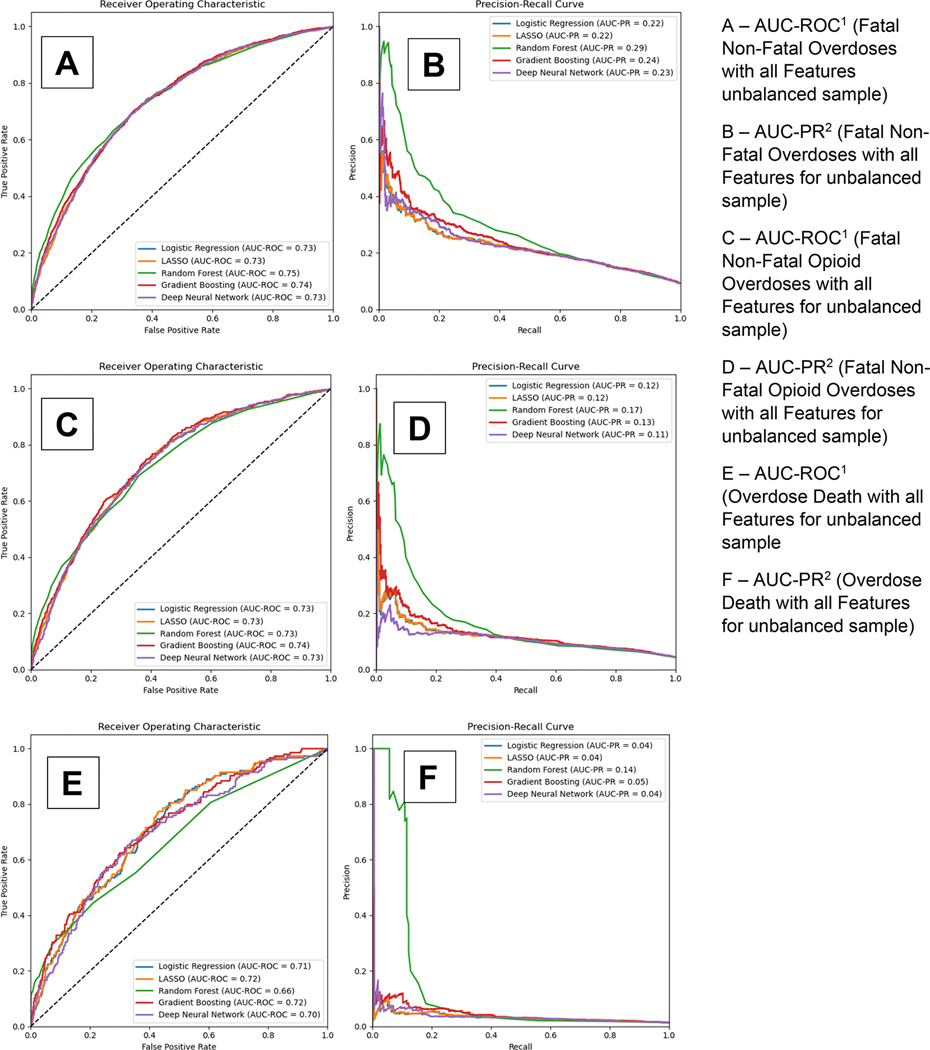

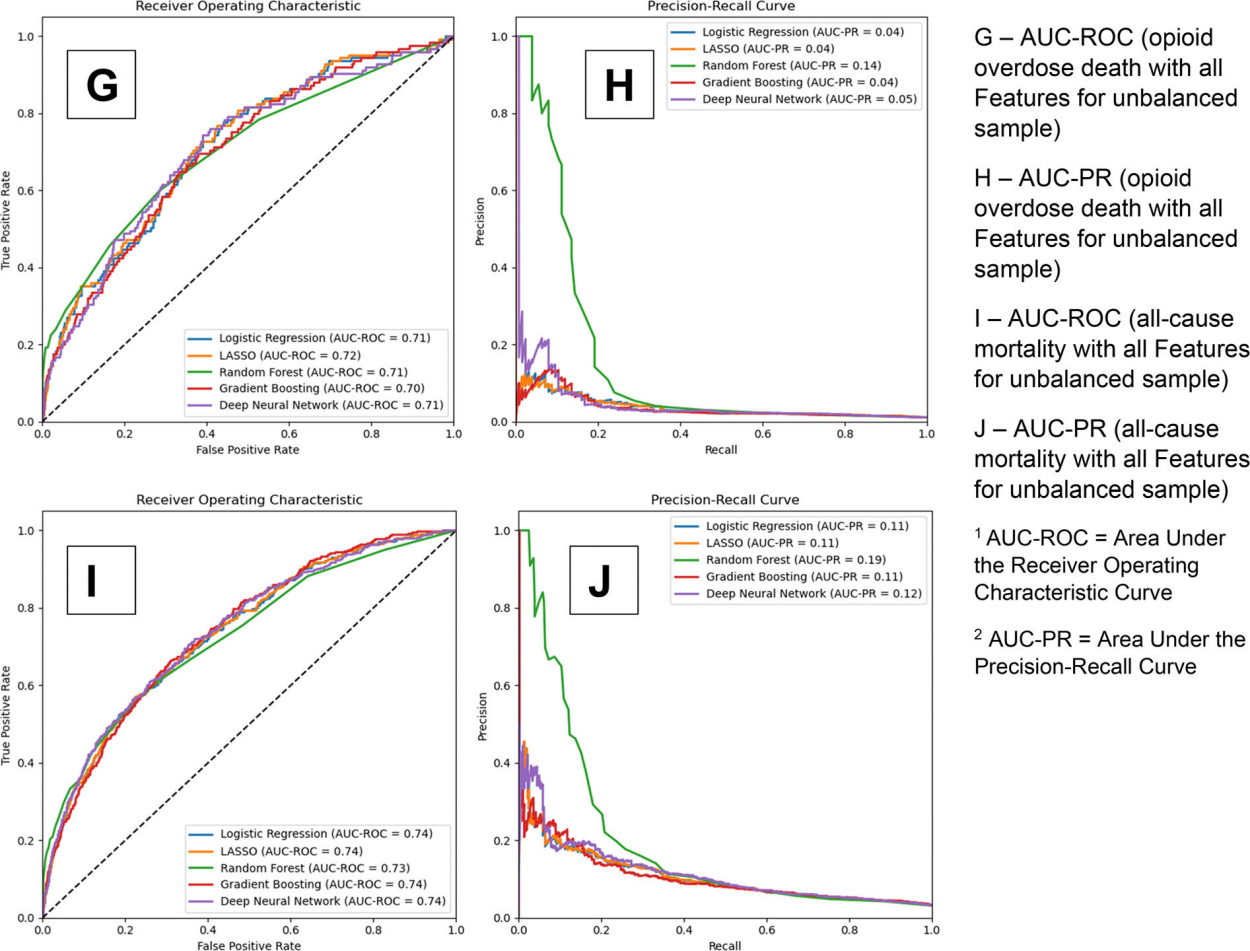
Area under the receiver operating curve and precision-recall curve for secondary outcomes among the unbalanced sample.

Figure 3 displays the AUC-ROC and precision-recall curves for our 5 models for the secondary outcomes of fatal and non-fatal overdoses, fatal and non-fatal opioid overdoses, overdose death, opioid overdose death, and all-cause mortality respectively. Panels A and B provide the curves for the models with all candidate predictors without a balancing technique for fatal and non-fatal overdoses. Panels C and D, E and F, G and H, and I and J provide the results for fatal and non-fatal opioid overdoses, overdose death, opioid overdose death, and all-cause mortality respectively. As with B-MOUD retention, GBM, RF, and DNN generally provided the highest levels of discrimination with the balancing techniques yielding slight to significant improvements in predictive performance (Fatal and Non-Fatal Opioid Overdoses—under-sampling: RF AUC-ROC: 0.77, GBM AUC-ROC: 0.76; Opioid Overdose Death—under-sampling: RF AUC-ROC: 0.79, DNN AUC-ROC: 0.78).

eTable 4 also provides the optimized sensitivity and specificity, based on the Youden index, for each of the secondary outcomes. For fatal and non-fatal overdoses among the unbalanced sample, GBM had an AUC-ROC of 0.74, a sensitivity of 71% and specificity of 65%, and RF had an AUC-ROC of 0.75, a sensitivity of 74% and specificity of 62%. For fatal and non-fatal opioid overdoses among the unbalanced sample, GBM had an AUC-ROC of 0.74, a sensitivity of 68% and specificity of 69%, and RF had an AUC-ROC of 0.74, a sensitivity of 65%, and specificity of 70%. For overdose death, opioid overdose death, and all-cause mortality among the unbalanced sample, GBM had an AUC-ROC of 0.71, 0.74 and 0.74 respectively and a sensitivity of 53%, 44%, and 64% and specificity of 76%, 85%, and 74% respectively.

eFigures 7–11 provide the most important predictors (*n* = 59 for fatal and non-fatal overdoses, *n* = 60 for fatal and non-fatal opioid overdoses, *n* = 61 for overdose death, *n* = 61 for opioid overdose death, and *n* = 60 for all-cause mortality; cut point for cumulative performance of >80%) for the unbalanced sample using the RF models. While the most influential predictors stay relatively constant, the order of these most important predictors change depending on the secondary outcome. No single predictor accounted for more than 3% of the model’s prediction of the secondary outcomes.

### Sensitivity analyses

Predictive performance metrics for the models using principal components were lower than those among the full candidate predictors (AUC-ROC ranged from 0.57 to 0.63). However, limiting the number of predictors to those with cumulative importance of >80% resulted in models with similar predictive performance metrics as the full models; accuracy decreased by approximately 1% (results available upon request).

## Discussion

Using national-level data from the VHA CDW, we developed and validated a suite of machine-learning models with moderate predictive performance for predicting B-MOUD retention, fatal and non-fatal overdoses, fatal and non-fatal opioid overdoses, overdose death, opioid overdose death, and all-cause mortality. For our primary outcome of B-MOUD retention, the GBM model performed similarly across the unbalanced and ROSE and under-sampling balancing techniques (0.72), likely because B-MOUD retention was not significantly unbalanced. The predictive performance for the secondary outcomes was similar across models in the unbalanced sample, also achieving moderate AUC-ROC (i.e., 0.7–0.8). Surprisingly, the DNN models either performed as well as or underperformed for all outcomes as compared to other machine-learning models, likely due to the limited number of B-MOUD episodes. DNN models generally require more data, compared to classical machine-learning models, given their complex nature. Application of SMOTE, ROSE, and under-sampling, which work best for weak classifers,^57^ improved the predictive performance of the models among the secondary outcomes where the prevalence was low (1%−9%); under-sampling, for example, improved the predictive performance of the RF model by 0.1 for opioid overdose death (increasing from 0.69 among the unbalanced sample to 0.79). Currently there are no formal methods in clinical practice to identify patients at elevated risk of B-MOUD discontinuation. These models have important potential for real-time use within the electronic health record by clinicians to identify patients newly initiated on B-MOUD that are at high risk of B-MOUD discontinuation and experiencing fatal and non-fatal overdose that may lead to clinician interventions that could mitigate harms in this vulnerable group of patients. Specifically, a decision support tool, based on this predictive model, could be created that could update nightly to identify patients at high risk of B-MOUD discontinuation. A clinician could use this tool to talk with the patient about their risk and implement strategies to help minimize that risk (e.g., more frequent follow-up, contingency management).

While many previous studies have identified predictors of treatment retention,^58–63^ we found only two models that have been previously published predicting treatment retention using Massachusetts All-Payer Claims Database^52^ and Treatment Episode Data Set—Discharges.^64^ Like our study, these models did not achieve an AUC-ROC > 0.8, a common minimal target if wanting to influence clinical decisions.^65,66^ Another prediction modeling study that used electronic health record data from 23 different substance use or mental health care programs predicted B-MOUD discontinuation in the 3 months post-initiation achieved recall of 75% and precision of 90%, but the number of patients in the cohort was limited (∼5,000).^63^ To our knowledge, no other studies have developed prediction models of the secondary outcomes used in this study (i.e., fatal and non-fatal overdoses, fatal and non-fatal opioid overdoses, overdose death, opioid overdose death, and all-cause mortality) among patients initiating MOUD for OUD. To the best of our knowledge, no studies have developed predictive models of our primary and secondary outcomes among U.S. military veterans.

Two important clinical implications can be drawn from this predictive modeling study. First, no predictor contributed more than 4% to the model’s prediction for any of the outcomes in this study. As many clinicians already know, retaining persons with OUD on MOUD is a complex issue; given that no one predictor alone contributes a substantial portion of the model’s prediction indicates that B-MOUD retention and overdose among this patient population is complex and simple heuristics that may be used by clinicians to identify likely discontinuation or overdose are unlikely to be adequate. Secondly, B-MOUD retention is a multi-faceted problem that will likely require a constelation of interventions targeting multiple predictive factors. For example, it may be that a suite of actions are needed to address the multiple predictors such as raising the B-MOUD dose, incorporating psychotherapy, or arranging transportation. In addition, these findings point to a real need for machine-learning approaches to help clinicians identify those at risk since no one predictor contributed significantly to the prediction. Our sensitivity analysis of limiting to only those predictors with a cumulative importance greater than 80% (roughly 55–60 predictors) decreased the accuracy of the models only by approximately 1%; therefore, the tradeoff between using all predictors and the limited number of predictors, when implementing in a real-time clinical decision support tool, is minimal in regard to accuracy loss but may cut down substantially the amount of computing time to derive the predictors. This would not affect the end-user clinician or patient but could help lower the information technology barriers to implementation.

### Limitations

This study should be interpreted in light of several important limitations. First, B-MOUD retention was measured using B-MOUD inpatient orders or outpatient pharmacy prescriptions/administrations that were dispensed from VHA or paid for by VHA. Therefore, receipt of B-MOUD from a non-VHA pharmacy that is not paid for by VHA will not be captured and may result in misclassification of B-MOUD retention. B-MOUD receipt can also be missed if a veteran is admitted to a non-VHA hospital and uses non-VHA insurance. Second, non-fatal overdoses, as used in our secondary outcomes, are likely under-represented in the CDW as non-fatal overdoses that occur at non-VHA facilities and are not paid for by VHA are not captured in the data. Relatedly, non-fatal overdoses that do not result in a healthcare visit are not captured in any data source. Third, deaths due to overdose can also be under-represented due to undercounting of drug involvement on death certificates. Recent estimates show that opioid overdose deaths are 20–35% higher each year than reported.^67^ Fourth, initiations of B-MOUD, despite using many years of data, are still relatively low. Therefore, more B-MOUD episodes among more veterans could be helpful for improving the predictive performance of our models, particularly for DNN. Future work will continue to refine the models as more data become available and explore the incorporation of data from other sources (e.g., unstructured data from clinical notes). Fifth, we did not require a minimum number of days of B-MOUD to constitute a B-MOUD episode which means some episodes may be from detoxification instead of true initiation of treatment. Sixth, this study evaluated B-MOUD episodes which are nested within individuals. Therefore, the validation statistics may be different if studying only the first B-MOUD episode per veteran. Seventh, Lagisetty et al. 2021,^24^ from which the ICD-10-CM definition for OUD was derived, noted the limitations of using ICD-9-CM/ICD-10-CM codes for identifying individuals with true OUD. Specifically, the authors note only 57.7% of veterans identified as having OUD per ICD-9-CM/ICD-10-CM codes were categorized as high likelihood of truly having OUD. Therefore, the use of any ICD-based definition of OUD may overestimate the number of veterans with OUD. Eighth, traditional machine learning approaches, such as those used in this study (e.g., random forest), do not provide information on the directional effects of predictor variables. Therefore, we cannot provide coefficients for the magnitude and direction of effect of a particular predictor on the outcome of interest.

## Conclusions

Through this study, we demonstrate the feasibility of developing and validating a suite of machine-learning algorithms to predict B-MOUD retention, fatal and non-fatal overdoses, and death among veterans initiating B-MOUD for OUD treatment. All of our models have moderate predictive performance. Future research should assess how these models can be improved with additional data sources (e.g., clinical notes). Future research should also evaluate how these models can be incorporated into an electronic health record-based clinical decision support tool identifying veterans at elevated risk of B-MOUD discontinuation and other important MOUD-related outcomes.

## Supplementary Material

eFigure 1

eFigure 2

eFigure 3

eFigure 5

eFigure 6

eFigure 4

eFigure 7

eFigure 8

eFigure 9

eTable 1

eFigure 10

eFigure 11

eTable 2

eTable 3

eTable 4

Supplemental data for this article can be accessed online at https://doi.org/10.1080/10550887.2024.2363035.

## Figures and Tables

**Table 1.** Outcome and sociodemographic characteristics among B-moud^1^ episodes divided into training and testing cohorts.

*N* = 56,547	Training^2^ (*N* = 45,237)	Testing^2^ (*N* = 11,310)

**Discontinued B-MOUD**^1^ **before 180 Days (N, %)**	17,006 (37.59%)	4,273 (37.78%)
**Experienced a Fatal or Non-Fatal Overdose (N, %)**	4,471 (9.88%)	1,035 (9.15%)
**Experienced a Fatal or Non-Fatal Opioid Overdose (N, %)**	2,208 (4.88%)	501 (4.43%)
**Died from an Overdose (N, %)**	587 (1.30%)	155 (1.37%)
**Died from an Opioid Overdose (N, %)**	475 (1.05%)	125 (1.11%)
**Died from any Cause (N, %)**	1,442 (3.19%)	355 (3.14%)
**Sex (N, %)**		
Male	42,065 (92.99%)	10,496 (92.80%)
**Race (N, %)**		
American Indian or Alaska Native	330 (0.73%)	76 (0.67%)
Asian	132 (0.29%)	29 (0.26%)
Black or African American	7,336 (16.21%)	1,848 (16.33%)
Native Hawaiian or Other Pacific Islander	248 (0.54%)	63 (0.55%)
White	35,300 (78.03%)	8,858 (78.32%)
More than one race	397 (0.88%)	79 (0.70%)
Declined to Answer	1,494 (3.30%)	357 (3.16%)
**Ethnicity (N, %)**		
Hispanic or Latino	2,392 (5.29%)	578 (5.11%)
Not Hispanic or Latino	41,917 (92.67%)	10,514 (92.96%)
Declined to Answer	928 (2.05%)	218 (1.93%)
**Age (Mean, SD**^3^**)**	46.59 (13.96)	46.36 (13.99)
**Baseline Employment Status (N, %)**		
Employed	8,755 (19.35%)	2,130 (18.83%)
Unemployed	32,701 (72.28%)	8,262 (73.05%)
Retired	3,781 (8.36%)	918 (8.12%)
**Baseline Marital Status (N, %)**		
Never Married	12,337 (27.29%)	3,107 (27.48%)
Married	11,690 (25.86%)	2,897 (25.62%)
Separated	3,843 (8.50%)	970 (8.58%)
Divorced	15,964 (35.31%)	4,039 (35.73%)
Widowed	1,371 (3.03%)	292 (2.58%)
**Baseline Priority Group (N, %)**		
1	18,650 (41.22%)	4,700 (44.14%)
2	3,039 (6.71%)	728 (6.84%)
3	4,319 (9.55%)	1,098 (10.31%)
4	3,147 (6.96%)	124 (1.16%)
5	12,982 (28.69%)	3,250 (30.52%)
6	722 (1.60%)	192 (1.80%)
7 A & 7 C	551 (1.22%)	124 (1.16%)
8 A-G	1,827 (4.04%)	430 (4.03%)
**Baseline Zip Code Rurality (N, %)**		
Urban	38,701 (85.55%)	9,745 (86.16%)
Large Rural City/Town	3,796 (8.39%)	929 (8.21%)
Isolated Small Rural Town	2,740 (6.06%)	636 (5.62%)
**Prior Year Social Risk (N, %)**		
Justice Involved	4,598 (10.16%)	1,196 (10.57%)
Unhoused	18,041 (39.88%)	4,577 (40.47%)
**Baseline CDC/ATSDR Social Vulnerability Index Score**		
Socioeconomic Status (Mean, SD^3^)	0.43 (0.24)	0.43 (0.24)
Household Composition and Disability (Mean, SD^3^)	0.35 (0.26)	0.35 (0.26)
Minority Status and Language (Mean, SD^3^)	0.72 (0.25)	0.74 (0.25)
Housing Type and Transportation (Mean, SD^3^)	0.63 (0.23)	0.63 (0.23)
Overall Vulnerability (Mean, SD^3^)	0.53 (0.24)	0.53 (0.24)
**Elixhauser Comorbidity Count (Mean, SD** ^3^ **)**	4.14 (2.36)	4.17 (2.34)
**Prior Year Diagnoses (N, %)**		
**Medical History**		
HCV^4^	11,954 (26.42%)	3,059 (27.04%)
**Mental Health History**		
Anxiety Disorders	25,697 (56.80%)	6,549 (57.90%)
Bipolar Disorder	7,119 (15.73%)	1,824 (16.12%)
Depression	31,854 (70.41%)	8,101 (71.63%)
PTSD^5^	20,860 (46.11%)	5,320 (47.04%)
**Substance Use Disorder History**		
Alcohol Use Disorder	20,285 (44.84%)	5,119 (45.26%)
Non-Opioid Drug Use Disorder	21,860 (48.32%)	5,582 (49.35%)
Tobacco Use Disorder	28,646 (63.32%)	7,335 (64.85%)
**Number of Days Covered of Non-Opioid Medications in the 365 Days Prior to Episode Start Date (Mean, SD)**		
Antidepressants	107.17 (110.35)	108.4 (110.47)
Benzodiazepines	25.02 (71.83)	24.02 (69.89)
Hypnotics and Non-Benzodiazepine Sedatives	19.22 (58.5)	19.56 (58.82)
Skeletal Muscle Relaxants	23.38 (58.98)	23.44 (58.77)
**Days’ Supply of B-MOUD**^1^ **on Episode Start Date (Mean, SD**^3^**)**	10.05 (12.92)	9.85 (12.78)
**Dose of B-MOUD on the Index Date (Mean, SD** ^3^ **)**	13.08 (14.65)	13.02 (15.33)
**Prior Year Healthcare Utilization**		
Number of Emergency Department Visits (Mean, SD^3^)	2.86 (4.87)	2.88 (4.90)
Number of Primary Care Visits (Mean, SD^3^)	12.54 (16.01)	12.67 (15.70)
Number of Inpatient Detoxification, General Psychiatry, or SUD Residential Treatment Inpatient Admissions (Mean, SD^3^)	0.86 (1.59)	0.91 (1.64)

1 B-MOUD = Buprenorphine treatment for opioid use disorder;

2 Training = Cohort for model training (80% of episodes), Testing = Cohort for model testing (20% of episodes)

3 SD = Standard deviation

4 HCV = Hepatitis C virus

5 PTSD = Post-traumatic stress disorder.

## Appendix

### Machine learning methods

For this study, we used five candidate machine-learning algorithms: (1) Multiple Logistic Regression (MLR), (2) Least Absolute Shrinkage and Selection Operator-Type Regression (LASSO) (3) Gradient Boosting Machine (GBM), (4) Random Forest (RF), and (5) Deep Neural Network (DNN). MLR, widely used in studies with binary outcomes, models the log odds of the outcome as a specified function of the predictor variables.^65^ MLR can have poor performance classifying the binary outcome when there are many predictors or multicollinearity. To address this limitation, we used a variant of MLR, LASSO regression that performs variable selection, thus allowing for a more parsimonious model and addressing multicollinearity. LASSO models use L1 regularization that results in some coefficient estimates being “shrunk” to exactly zero, thus de-selecting these variables from the model.^68^ Both MLR and LASSO regression require the analyst to specify a parametric form for the model, which can lead to poor model performance if the model form is incorrect. To address this limitation to parametric regression, we used two alternative algorithms, RF and GBM, that combine non-parametric decision tree classifiers to optimize outcome classification and a deep learning algorithm (DNN). RF is an ensemble decision tree-based algorithm that builds numerous decision trees in parallel to classify the outcomes and outputs the mode of the classes (classification) of the individual trees for a given input. RF is a flexible algorithm that can handle many predictor variables without making strong parametric assumptions about model form and prevents overfitting by building many decision trees and combining their outputs.^69,70^ GBM is an ensemble learning technique that sequentially combines multiple decision trees.^71^ While both RF and GBM are ensemble decision tree methods, RF independently constructs large numbers of shallow trees while GBM is trained sequentially, building trees one at a time, correcting errors of the prior tree. For this study, we used the ‘Gradient BoostingClassifer’ from the scikit-learn library in Python. DNN is a “deep learning” alternative to the algorithms described above. “Deep learning” refers to the use of multiple layers that extract higher-level features from the candidate predictors. DNN models form an interconnected network from the layers, which allows for interpretation of complex patterns in the data. DNNs adaptively learn from data by iteratively refining their parameters to minimize predicted error through procedures like backpropagation and utilizing various optimization approaches.^72^

### Hyperparameter tuning

We used the GridSearchCV technique for hyperparameter tuning.^49^ GridSearchCV provides each model hyperparameter with a range of possible values. The method uses cross-validation to fit the model, assess how well it works, and repeats the steps for each combination of these hyperparameter values. Cross-validation is a technique for assessing how the results of a model will generalize to an independent dataset. The dataset is split into two groups, a single of which is used for training the model and the other for testing it. This process is carried out multiple times using different data chunks. With this approach, the model’s optimum performance is determined by systematically finding the predefined hyperparameters. Below are the chosen hyperparameters for each model type.

### Multiple logistic Regression (MLR)

StandardScaler was used to normalize the candidate predictors before implementing MLR.^73^ For robustness in high-dimensional data settings, the MLR model was set up to iterate 10,000 times (**max_iter = 10000**) to ensure convergence.

### Random Forest (RF)

RF is well-known for its effectiveness in managing intricate relationships and non-linear interactions. For hyperparameter tuning, the number of trees in the forest (n_estimator), maximum depth of each tree (max_depth), the minimum number of samples required to split an internal node (min_sample_split), minimum number of samples required to be at a leaf node (min_samples_leaf) were considered. We selected hyperparamaters based on the values that maximized the AUC-ROC. Specifically, we set the maximum depth of the trees at 30 (**max_depth = 30**) as this allowed for a balance between overfitting risk and model complexity. Two (**min_samples_split = 2**) and four samples (**min_samples_leaf = 4**) were the minimal numbers used to split an internal node and reach a leaf node, respectively. This arrangement enhances the model’s accuracy and helps improve the decision rules. A reliable aggregated prediction could be made with 300 trees in the ensemble (i.e., **n_estimators = 300**).

### Gradient boosting machine (GBM)

For GBM, the hyperparameters considered were number of boosting stages to be run (n_estimators), shrinks the contribution of each tree (learning_rate), maximum depth of the individual regression estimators (max_depth), the minimum number of samples required to split an internal node (min_sample_split), and the minimum number of samples required to be at a leaf node (min_samples_leaf). Like with RF, the hyperparamaters were selected based on the values that maximized the AUC-ROC. Specifically, we set the learning rate to 0.2 (**learning_rate = 0.2**), which balances learning accuracy and speed. To avoid overfitting, the maximum depth of each regression estimator was restricted to 3 (**max_depth = 3**). The model was set up with the least number of samples needed at a leaf node (**min_samples_leaf = 1**) and the minimum number of samples required to split a node (**min_samples_split = 2**), respectively. This architecture ensures a fine-grained approach to learning from the data. Two hundred trees (**n_estimators = 200**) made up the ensemble, which balanced computational effectiveness and model complexity.

### Deep neural network (DNN)

We tuned the DNN’s architecture and training procedure to maximize performance while reducing the possibility of overfitting. The number of features in the training dataset represents the dimensionality of the input that the first layer, a dense layer with 64 neurons, receives. This layer’s neurons use the Rectified Linear Unit (ReLU) activation function, which adds non-linearity to the model and helps it recognize more intricate patterns in the input. A dropout layer, which has a dropout rate of 0.5, is added after the first dense layer. By lessening the model’s sensitivity to certain weights, this layer helps avoid overfitting by arbitrarily setting a percentage of input units to 0 at each training update. The following layer is also dense and uses the ReLU activation function; it has 32 neurons. The modified inputs from the preceding levels are further processed in this layer. This dropout layer gives the model an extra degree of regularization by having a dropout rate of 0.5, much like the second layer. The final layer uses the sigmoid function and is dense, consisting of a single neuron. This setup works especially well for binary classification problems, such as those we were trying to model, since the output of the sigmoid function is a value between 0 and 1, which indicates the likelihood that the input belongs to one of the two classes.

### Methods for addressing imbalanced data

We used three techniques for reducing inherent imbalances in the datasets and assist model-building with improved prediction accuracy and generalization capacities. All three were used to be able to compare how each model performs with each technique. These techniques are (1) Synthetic Minority Over-Sampling Technique (SMOTE), (2) under-sampling, and (3) Random Over-Sampling Examples (ROSE). SMOTE aims to balance the class distribution by generating synthetic instances of the minority class, thereby enhancing the model’s ability to generalize well to unseen data.^74^ Under-sampling is a technique designed to adjust the class distribution by randomly eliminating some of the instances from the majority class. This helps in mitigating the dominance of the majority class and allows the model to perform better on minority instances.^75^ ROSE is an advanced over-sampling technique that generates synthetic instances of the minority class by considering the feature space’s distribution. This method not only increases the minority class instances but also introduces variability, aiding the model in better understanding and classifying the minority class.^76^ Several studies have shown these techniques improve prediction results.^77–81^
