# Supplementary figures and images for: Development and validation of machine-learning algorithms predicting retention, overdoses, and all-cause mortality among US military veterans treated with buprenorphine for opioid use disorder
Source: J Addict Dis. Author manuscript; Available in PMC 2026 Apr 7. (PMC13056003; doi:10.1080/10550887.2024.2363035)

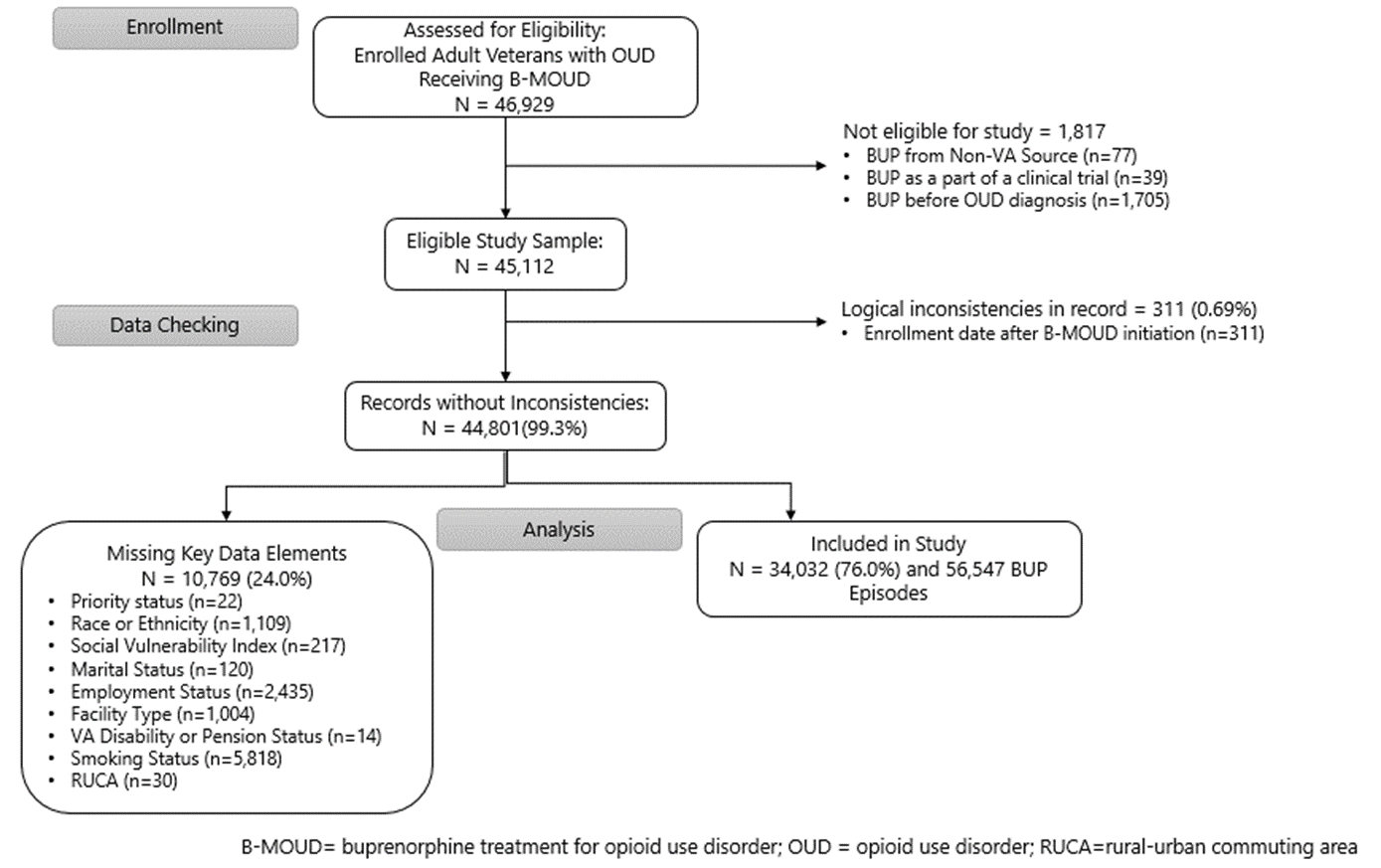


# eFigure 2. Derivation of the Study Cohort

Supplement: eFigure 2 [file NIHMS2063158-supplement-eFigure_2.docx]

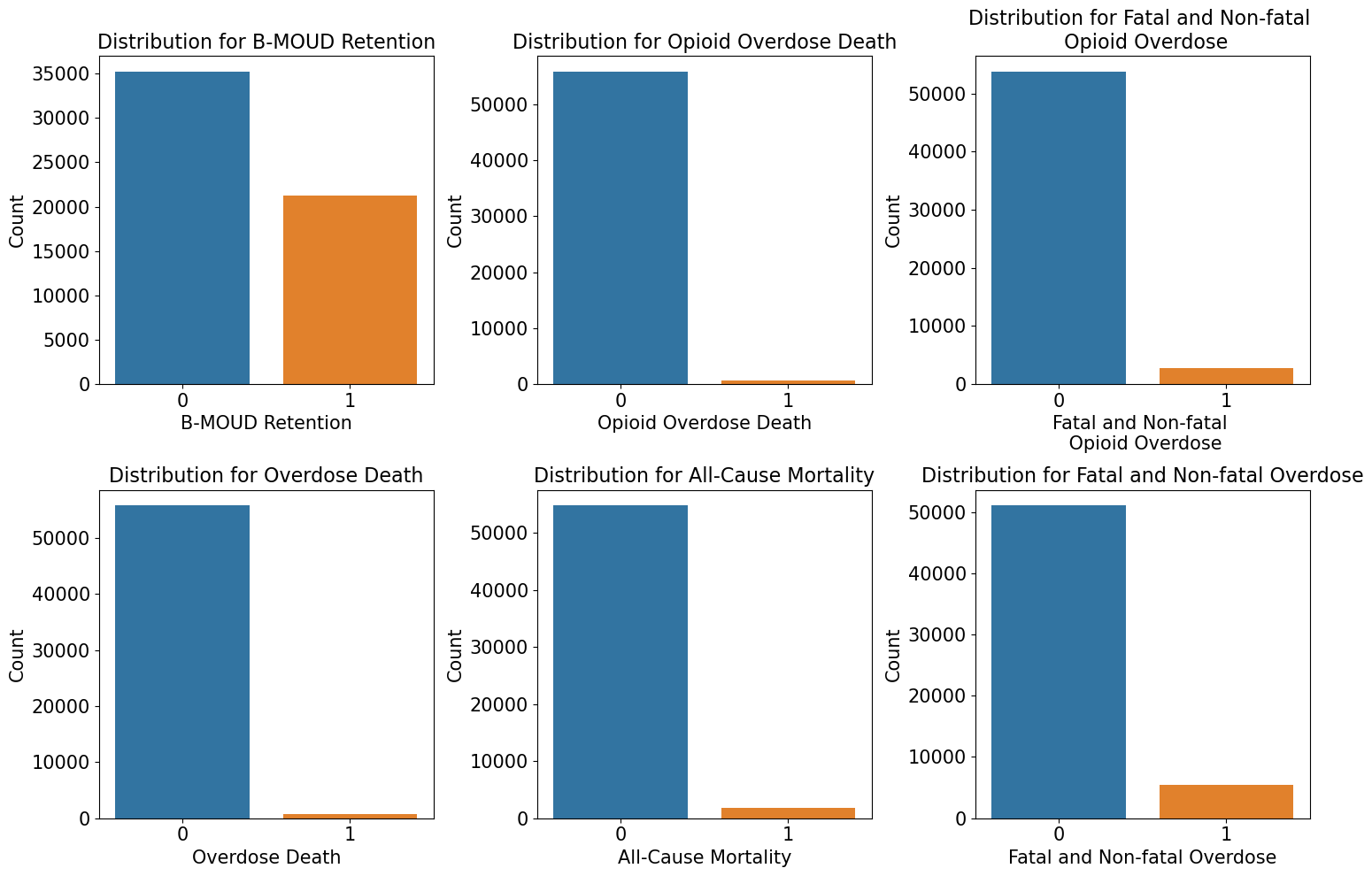


# eFigure 3: Outcome Class Distribution Before Balancing the Outcome

Supplement: eFigure 3 [file NIHMS2063158-supplement-eFigure_3.docx]
