# Supplementary material for: Development and validation of machine-learning algorithms predicting retention, overdoses, and all-cause mortality among US military veterans treated with buprenorphine for opioid use disorder
Source: J Addict Dis. Author manuscript; Available in PMC 2026 Apr 7. (PMC13056003; doi:10.1080/10550887.2024.2363035)
Supplement: eTable 1 [file NIHMS2063158-supplement-eTable_1.docx]

# eTable 1. Included OUD Diagnosis Codes

| **Diagnosis** | **Codes** |
| --- | --- |
| Opioid abuse | *ICD-9-CM:*  305.5- ^1^  *ICD-10-CM:*  F11.10, F11.12–, F11.14, F11.15–, F11.18–, F11.19 |
| Opioid dependence | *ICD-9-CM:*  304.00^1^, 304.01^1^, 304.02^1^, 304.7–^2^  *ICD-10-CM:*  F11.2–– |
| Opioid use | F11.9–– |
| ^1^ Note that the ICD-9-CM system lacked the specificity of ICD-10-CM. This code covers all morphine-type dependencies, including heroin, methadone, and opium along with all synthetic derivatives.  ^2^ Notes opioid use combined with other drug dependence, a smaller subset of cases located with the ICD-10-CM coding. | |
