# Supplementary material for: Development and validation of machine-learning algorithms predicting retention, overdoses, and all-cause mortality among US military veterans treated with buprenorphine for opioid use disorder
Source: J Addict Dis. Author manuscript; Available in PMC 2026 Apr 7. (PMC13056003; doi:10.1080/10550887.2024.2363035)
Supplement: eFigure 11 [file NIHMS2063158-supplement-eFigure_11.docx]

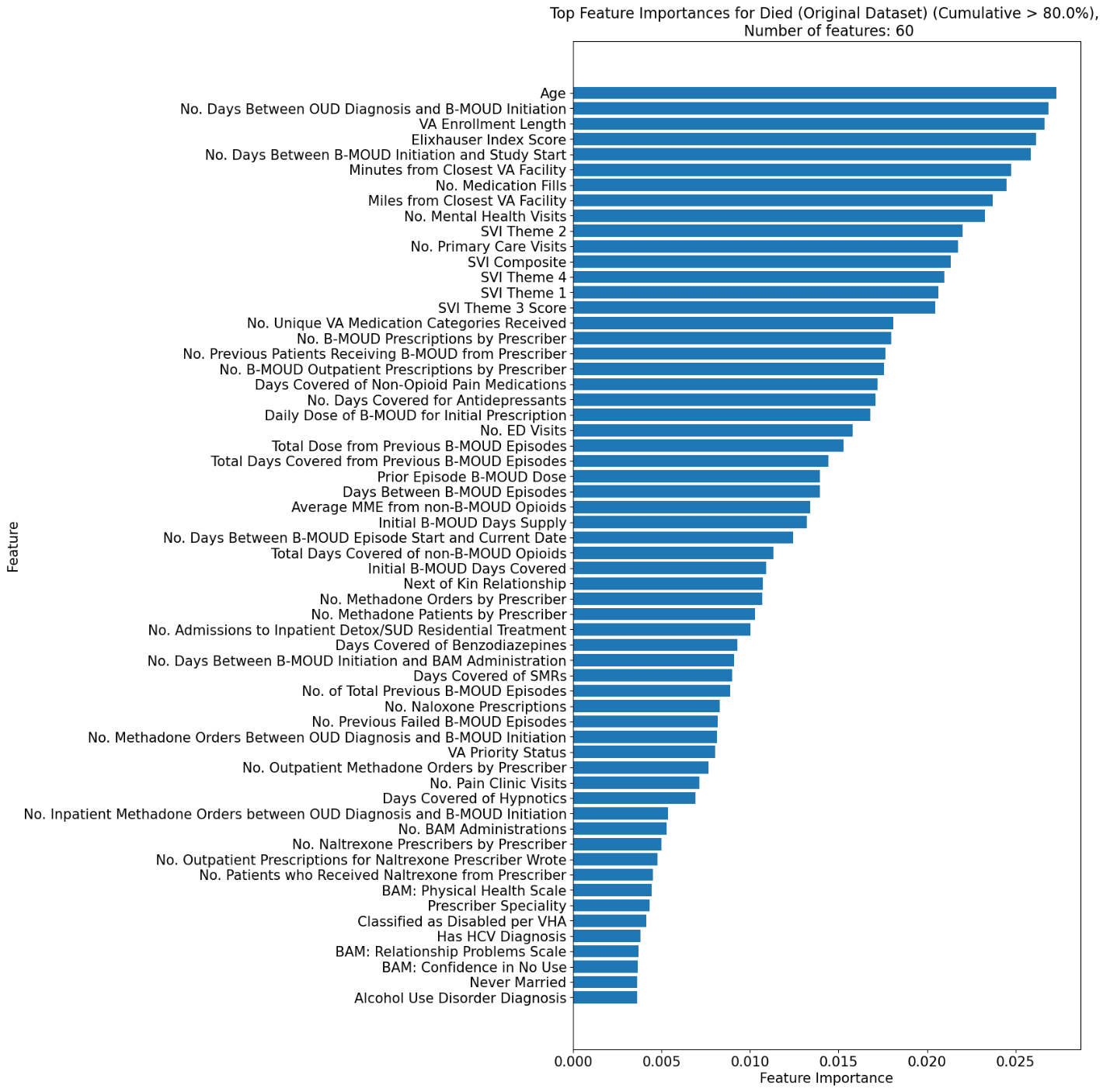


# eFigure 11. Predictors of Most Importance for All-Cause Mortality from the Random Forest Model among the Unbalanced Sample (n=60)
