# Supplementary material for: Development and validation of machine-learning algorithms predicting retention, overdoses, and all-cause mortality among US military veterans treated with buprenorphine for opioid use disorder
Source: J Addict Dis. Author manuscript; Available in PMC 2026 Apr 7. (PMC13056003; doi:10.1080/10550887.2024.2363035)
Supplement: eTable 2 [file NIHMS2063158-supplement-eTable_2.docx]

# eTable 2. Candidate Predictors for machine-learning models

| **Sociodemographic Factors** | **Medication Treatment for Opioid Use Disorder (MOUD) Profile Characteristics** | **Health Care Service Utilization** | **Disease Comorbidity Factors** |
| --- | --- | --- | --- |
| - Age - Gender - Length of time for Veterans Health Administration (VHA) enrollment - Religion - Race - Ethnicity - Marital status - VHA priority status - State of residence - U.S. Census Division of Residence - Rural-urban commuting area code - Miles from the closest VHA facility - Minutes of drive-time to the closest VHA facility - Employment status - VHA disability status - VHA service-connected disability eligibility status - VHA pension eligibility status - Smoking status - Next of kin (e.g., spouse, brother) | - Days’ supply of initial buprenorphine (BUP) - Dose of initial BUP - Days covered of initial BUP - Inpatient or outpatient initiation of BUP - Days between opioid use disorder (OUD) diagnosis and BUP initiation - Year of BUP initiation - Month of BUP initiation - Methadone orders between OUD diagnosis and BUP initiation - Days’ supply of methadone between OUD diagnosis and BUP initiation - Naltrexone orders between OUD diagnosis and BUP initiation - Days’ supply of naltrexone between OUD diagnosis and BUP initiation - Number of previous BUP episodes - Number of previous failed BUP episodes - Days covered from previous BUP episodes - Total BUP dose from previous BUP episodes - Average BUP dose from previous BUP episodes - Time since last BUP episode - Binary indicator for whether the difference between BUP initiation and start of data is 365 days - Fractional value for number of days between BUP initiation and fiscal year 2006 - Number of days between BUP initiation and start of fiscal year 2006 | - Number of emergency room visits - Number of primary care/medicine visits - Number of mental health visits - Number of pain clinic visits - Whether or not Brief Addiction Monitor (BAM) or Brief Addiction Monitor-Revised (BAM-R) has been administered - Number of BAM or BAM-R administrations - Days between BUP initiation and BAM administration - Individual constructs within BAM measured over the past 30 days (Days of sleep problems, Days of risky situations, Days of alcohol use, Days of other drug use, Days spent at work or school, Days of heavy alcohol use, Days of contact with a supportive person, Days distressed, Spiritual support, Confidence in no use, Physical health, Adequate income, Relationship problems, Urges/Cravings) | - Cannabis use disorder - Cocaine use disorder - Tobacco use disorder - Alcohol use disorder - Non-opioid, drug use disorder - Endocarditis - Hepatitis B Virus (HBV) - Hepatitis C Virus (HCV) - Human Immunodeficiency Virus (HIV) - Post-traumatic Stress Disorder (PTSD) - Opioid overdose - Non-opioid overdose - Any mental health disorder - Depression - Anxiety - Bipolar - Psychosis - Suicide or Self-inflicted Injury - Elixhauser Index |
