# Supplementary material for: Development and validation of machine-learning algorithms predicting retention, overdoses, and all-cause mortality among US military veterans treated with buprenorphine for opioid use disorder
Source: J Addict Dis. Author manuscript; Available in PMC 2026 Apr 7. (PMC13056003; doi:10.1080/10550887.2024.2363035)
Supplement: eTable 3 [file NIHMS2063158-supplement-eTable_3.docx]

# eTable 3. Candidate predictors for machine-learning models (continued)

| **Opioid-Specific Historical Factors** | **Non-Opioid Prescription Medication Utilization Factors** | **Social Risk Factors** | **Provider-Level Factors** | **Facility-Level Factors** |
| --- | --- | --- | --- | --- |
| - Opioid prescription duration of action - Opioid prescription schedule - Average morphine milligram equivalents (MME) - Total opioid days covered | - Number of medication fills received - Number of unique VHA categories of medications received - Number of naloxone prescriptions received - Days covered of antidepressants - Days covered of benzodiazepines - Days covered of skeletal muscle relaxants - Days covered of non-opioid analgesics - Days covered of hypnotics | - Social Vulnerability Index (SVI) Composite Score - SVI Household Composition & Disability Score - SVI Minority Status & Language Score - SVI Housing Type & Transportation Score - SVI Socioeconomic Score - Homelessness - Justice-Involvement | - Provider specialty - Provider credentials - VHA or non-VHA prescriber - Trainee - Number of BUP prescriptions written by prescriber - Number of methadone prescriptions written by prescriber - Number of naltrexone prescriptions written by prescriber - Number of patients prescriber wrote BUP for - Number of patients prescriber wrote methadone for - Number of patients prescriber wrote naltrexone for - Percent of patients retained on BUP in the year prior by prescriber | - Whether veteran received BUP from closest VHA facility - Facility type - Whether facility had an inpatient detoxification program - Whether facility had a methadone clinic - Whether facility had a SUD residential treatment program - Flag for VHA station |
