# Supplementary material for: Development and validation of machine-learning algorithms predicting retention, overdoses, and all-cause mortality among US military veterans treated with buprenorphine for opioid use disorder
Source: J Addict Dis. Author manuscript; Available in PMC 2026 Apr 7. (PMC13056003; doi:10.1080/10550887.2024.2363035)
Supplement: eTable 4 [file NIHMS2063158-supplement-eTable_4.docx]

# eTable 4. Predictive Performance for Primary and Secondary Outcomes Across Various Machine Learning Methods at the Optimized Threshold of the Youden Index

| **Outcomes/Methods** | **AUC-ROC^1^** | **Specificity** | **Sensitivity** | **PPV^2^** | **NPV^3^** | **F1 Score** | **Optimal Threshold** | **Youden Index** |
| --- | --- | --- | --- | --- | --- | --- | --- | --- |
| **Primary Outcome** | | | | | | | | |
| **Retention (Unbalanced)** |  |  |  |  |  |  |  |  |
| MLR | 0.69 | 0.55 | 0.72 | 0.49 | 0.72 | 0.58 | 0.36 | 0.27 |
| LASSO | 0.69 | 0.55 | 0.72 | 0.49 | 0.72 | 0.58 | 0.36 | 0.27 |
| RF | 0.71 | 0.55 | 0.75 | 0.52 | 0.68 | 0.59 | 0.33 | 0.3 |
| GBM | 0.72 | 0.54 | 0.78 | 0.53 | 0.69 | 0.6 | 0.33 | 0.32 |
| DNN | 0.69 | 0.57 | 0.70 | 0.5 | 0.71 | 0.59 | 0.38 | 0.28 |
| **Retention (SMOTE)** |  |  |  |  |  |  |  |  |
| MLR | 0.66 | 0.56 | 0.68 | 0.48 | 0.68 | 0.56 | 0.38 | 0.24 |
| LASSO | 0.66 | 0.56 | 0.68 | 0.48 | 0.68 | 0.56 | 0.38 | 0.24 |
| RF | 0.71 | 0.54 | 0.77 | 0.51 | 0.77 | 0.61 | 0.39 | 0.31 |
| GBM | 0.71 | 0.60 | 0.71 | 0.52 | 0.71 | 0.60 | 0.38 | 0.31 |
| DNN | 0.70 | 0.58 | 0.71 | 0.50 | 0.71 | 0.59 | 0.38 | 0.28 |
| **Retention (ROSE)** |  |  |  |  |  |  |  |  |
| MLR | 0.69 | 0.55 | 0.72 | 0.49 | 0.72 | 0.58 | 0.48 | 0.27 |
| LASSO | 0.69 | 0.55 | 0.72 | 0.49 | 0.72 | 0.58 | 0.48 | 0.27 |
| RF | 0.72 | 0.61 | 0.71 | 0.53 | 0.71 | 0.61 | 0.48 | 0.33 |
| GBM | 0.72 | 0.60 | 0.73 | 0.52 | 0.73 | 0.61 | 0.48 | 0.33 |
| DNN | 0.69 | 0.66 | 0.62 | 0.53 | 0.61 | 0.57 | 0.42 | 0.28 |
| **Retention (Under Sampling)** |  |  |  |  |  |  |  |  |
| MLR | 0.68 | 0.59 | 0.66 | 0.62 | 0.66 | 0.64 | 0.52 | 0.26 |
| LASSO | 0.68 | 0.59 | 0.67 | 0.62 | 0.67 | 0.64 | 0.52 | 0.26 |
| RF | 0.71 | 0.60 | 0.71 | 0.63 | 0.71 | 0.67 | 0.50 | 0.31 |
| GBM | 0.72 | 0.66 | 0.66 | 0.66 | 0.66 | 0.66 | 0.54 | 0.32 |
| DNN | 0.69 | 0.60 | 0.68 | 0.62 | 0.68 | 0.65 | 0.53 | 0.27 |
| **Secondary Outcomes** | | | | | | | | |
| **Fatal and Non-Fatal Overdoses (Unbalanced)** |  |  |  |  |  |  |  |  |
| MLR | 0.73 | 0.67 | 0.69 | 0.17 | 0.69 | 0.28 | 0.09 | 0.36 |
| LASSO | 0.73 | 0.67 | 0.69 | 0.17 | 0.69 | 0.28 | 0.09 | 0.36 |
| RF | 0.75 | 0.62 | 0.74 | 0.17 | 0.74 | 0.27 | 0.1 | 0.37 |
| GBM | 0.74 | 0.65 | 0.71 | 0.17 | 0.71 | 0.27 | 0.08 | 0.36 |
| DNN | 0.69 | 0.57 | 0.71 | 0.50 | 0.71 | 0.59 | 0.38 | 0.28 |
| **Fatal and Non-Fatal Overdoses (SMOTE)** |  |  |  |  |  |  |  |  |
| MLR | 0.63 | 0.70 | 0.49 | 0.14 | 0.49 | 0.22 | 0.17 | 0.19 |
| LASSO | 0.63 | 0.68 | 0.51 | 0.14 | 0.51 | 0.22 | 0.16 | 0.19 |
| RF | 0.72 | 0.63 | 0.70 | 0.16 | 0.70 | 0.26 | 0.21 | 0.33 |
| GBM | 0.69 | 0.62 | 0.66 | 0.15 | 0.66 | 0.24 | 0.11 | 0.27 |
| DNN | 0.72 | 0.65 | 0.69 | 0.17 | 0.69 | 0.27 | 0.12 | 0.34 |
| **Fatal and Non-Fatal Overdoses (ROSE)** |  |  |  |  |  |  |  |  |
| MLR | 0.73 | 0.68 | 0.68 | 0.18 | 0.68 | 0.28 | 0.48 | 0.36 |
| LASSO | 0.73 | 0.68 | 0.68 | 0.18 | 0.68 | 0.28 | 0.48 | 0.36 |
| RF | 0.76 | 0.71 | 0.69 | 0.19 | 0.69 | 0.30 | 0.22 | 0.39 |
| GBM | 0.74 | 0.63 | 0.72 | 0.16 | 0.72 | 0.27 | 0.41 | 0.35 |
| DNN | 0.74 | 0.65 | 0.71 | 0.17 | 0.70 | 0.27 | 0.38 | 0.36 |
| **Fatal and Non-Fatal Overdoses (Under Sampling)** |  |  |  |  |  |  |  |  |
| MLR | 0.75 | 0.63 | 0.75 | 0.66 | 0.75 | 0.70 | 0.46 | 0.38 |
| LASSO | 0.75 | 0.62 | 0.76 | 0.66 | 0.76 | 0.70 | 0.45 | 0.38 |
| RF | 0.77 | 0.62 | 0.79 | 0.66 | 0.79 | 0.72 | 0.47 | 0.41 |
| GBM | 0.76 | 0.6 | 0.76 | 0.65 | 0.80 | 0.72 | 0.43 | 0.4 |
| DNN | 0.76 | 0.6 | 0.79 | 0.65 | 0.79 | 0.72 | 0.49 | 0.4 |
| **Fatal and Non-Fatal Opioid Overdoses (Unbalanced)** |  |  |  |  |  |  |  |  |
| MLR | 0.73 | 0.63 | 0.72 | 0.08 | 0.72 | 0.15 | 0.04 | 0.35 |
| LASSO | 0.73 | 0.63 | 0.72 | 0.08 | 0.72 | 0.15 | 0.04 | 0.35 |
| RF | 0.74 | 0.7 | 0.65 | 0.09 | 0.65 | 0.16 | 0.06 | 0.34 |
| GBM | 0.74 | 0.69 | 0.68 | 0.09 | 0.68 | 0.16 | 0.04 | 0.37 |
| DNN | 0.73 | 0.59 | 0.80 | 0.08 | 0.80 | 0.14 | 0.03 | 0.35 |
| **Fatal and Non-Fatal Opioid Overdoses (SMOTE)** |  |  |  |  |  |  |  |  |
| MLR | 0.61 | 0.45 | 0.71 | 0.06 | 0.71 | 0.11 | 0.04 | 0.16 |
| LASSO | 0.61 | 0.45 | 0.71 | 0.06 | 0.71 | 0.11 | 0.04 | 0.16 |
| RF | 0.72 | 0.70 | 0.63 | 0.09 | 0.63 | 0.16 | 0.16 | 0.33 |
| GBM | 0.68 | 0.65 | 0.61 | 0.08 | 0.61 | 0.13 | 0.07 | 0.26 |
| DNN | 0.70 | 0.52 | 0.77 | 0.07 | 0.77 | 0.13 | 0.04 | 0.30 |
| **Fatal and Non-Fatal Opioid Overdoses (ROSE)** |  |  |  |  |  |  |  |  |
| MLR | 0.73 | 0.69 | 0.66 | 0.08 | 0.66 | 0.16 | 0.48 | 0.35 |
| LASSO | 0.73 | 0.69 | 0.66 | 0.08 | 0.66 | 0.16 | 0.48 | 0.35 |
| RF | 0.77 | 0.75 | 0.64 | 0.11 | 0.64 | 0.18 | 0.14 | 0.39 |
| GBM | 0.74 | 0.68 | 0.69 | 0.09 | 0.69 | 0.16 | 0.39 | 0.37 |
| DNN | 0.73 | 0.64 | 0.71 | 0.08 | 0.71 | 0.15 | 0.23 | 0.35 |
| **Fatal and Non-Fatal Opioid Overdoses (Under Sampling)** |  |  |  |  |  |  |  |  |
| MLR | 0.73 | 0.68 | 0.67 | 0.66 | 0.67 | 0.68 | 0.48 | 0.35 |
| LASSO | 0.73 | 0.68 | 0.67 | 0.66 | 0.67 | 0.68 | 0.48 | 0.35 |
| RF | 0.74 | 0.66 | 0.69 | 0.66 | 0.7 | 0.68 | 0.49 | 0.36 |
| GBM | 0.73 | 0.63 | 0.71 | 0.64 | 0.71 | 0.67 | 0.45 | 0.34 |
| DNN | 0.73 | 0.71 | 0.65 | 0.68 | 0.65 | 0.66 | 0.52 | 0.36 |
| **Overdose Death (Unbalanced)** |  |  |  |  |  |  |  |  |
| MLR | 0.71 | 0.60 | 0.70 | 0.02 | 0.70 | 0.05 | 0.01 | 0.31 |
| LASSO | 0.72 | 0.60 | 0.72 | 0.02 | 0.72 | 0.05 | 0.01 | 0.32 |
| RF | 0.70 | 0.65 | 0.66 | 0.02 | 0.66 | 0.04 | 0.01 | 0.31 |
| GBM | 0.71 | 0.76 | 0.53 | 0.03 | 0.53 | 0.06 | 0.01 | 0.3 |
| DNN | 0.70 | 0.82 | 0.44 | 0.03 | 0.44 | 0.06 | 0.01 | 0.26 |
| **Overdose Death (SMOTE)** |  |  |  |  |  |  |  |  |
| MLR | 0.57 | 0.59 | 0.54 | 0.02 | 0.54 | 0.03 | 0.02 | 0.13 |
| LASSO | 0.57 | 0.60 | 0.52 | 0.02 | 0.52 | 0.03 | 0.02 | 0.12 |
| RF | 0.70 | 0.59 | 0.69 | 0.02 | 0.69 | 0.04 | 0.06 | 0.28 |
| GBM | 0.62 | 0.82 | 0.35 | 0.03 | 0.35 | 0.05 | 0.06 | 0.18 |
| DNN | 0.66 | 0.63 | 0.63 | 0.02 | 0.63 | 0.04 | 0.01 | 0.26 |
| **Overdose Death (ROSE)** |  |  |  |  |  |  |  |  |
| MLR | 0.72 | 0.52 | 0.82 | 0.02 | 0.82 | 0.04 | 0.35 | 0.33 |
| LASSO | 0.72 | 0.53 | 0.81 | 0.02 | 0.81 | 0.05 | 0.36 | 0.34 |
| RF | 0.74 | 0.79 | 0.57 | 0.04 | 0.57 | 0.07 | 0.05 | 0.35 |
| GBM | 0.73 | 0.70 | 0.66 | 0.03 | 0.66 | 0.06 | 0.27 | 0.36 |
| DNN | 0.70 | 0.93 | 0.27 | 0.05 | 0.27 | 0.09 | 0.01 | 0.20 |
| **Overdose Death (Under Sampling)** |  |  |  |  |  |  |  |  |
| MLR | 0.71 | 0.50 | 0.82 | 0.67 | 0.82 | 0.74 | 0.31 | 0.32 |
| LASSO | 0.72 | 0.48 | 0.85 | 0.67 | 0.85 | 0.75 | 0.29 | 0.33 |
| RF | 0.73 | 0.80 | 0.55 | 0.78 | 0.55 | 0.65 | 0.54 | 0.36 |
| GBM | 0.74 | 0.62 | 0.73 | 0.70 | 0,73 | 0.72 | 0.38 | 0.38 |
| DNN | 0.68 | 0.66 | 0.62 | 0.69 | 0.62 | 0.65 | 0.48 | 0.28 |
| **Opioid Overdose Death (Unbalanced)** |  |  |  |  |  |  |  |  |
| MLR | 0.71 | 0.69 | 0.59 | 0.02 | 0.59 | 0.04 | 0.01 | 0.28 |
| LASSO | 0.72 | 0.69 | 0.60 | 0.02 | 0.60 | 0.04 | 0.01 | 0.29 |
| RF | 0.69 | 0.70 | 0.60 | 0.02 | 0.61 | 0.04 | 0.01 | 0.31 |
| GBM | 0.74 | 0.85 | 0.44 | 0.03 | 0.44 | 0.06 | 0.01 | 0.29 |
| DNN | 0.71 | 0.84 | 0.39 | 0.02 | 0.39 | 0.05 | 0.01 | 0.23 |
| **Opioid Overdose Death (SMOTE)** |  |  |  |  |  |  |  |  |
| MLR | 0.57 | 0.64 | 0.50 | 0.02 | 0.50 | 0.03 | 0.02 | 0.14 |
| LASSO | 0.57 | 0.32 | 0.50 | 0.02 | 0.50 | 0.03 | 0.02 | 0.14 |
| RF | 0.70 | 0.80 | 0.47 | 0.03 | 0.47 | 0.05 | 0.08 | 0.28 |
| GBM | 0.58 | 0.66 | 0.56 | 0.02 | 0.56 | 0.03 | 0.02 | 0.22 |
| DNN | 0.70 | 0.74 | 0.55 | 0.02 | 0.55 | 0.05 | 0.01 | 0.30 |
| **Opioid Overdose Death (ROSE)** |  |  |  |  |  |  |  |  |
| MLR | 0.71 | 0.49 | 0.86 | 0.02 | 0.86 | 0.04 | 0.30 | 0.34 |
| LASSO | 0.71 | 0.47 | 0.86 | 0.02 | 0.86 | 0.04 | 0.30 | 0.34 |
| RF | 0.73 | 0.67 | 0.67 | 0.02 | 0.67 | 0.04 | 0.03 | 0.35 |
| GBM | 0.71 | 0.70 | 0.64 | 0.02 | 0.64 | 0.04 | 0.22 | 0.34 |
| DNN | 0.70 | 0.99 | 0.19 | 0.15 | 0.19 | 0.17 | 0.14 | 0.18 |
| **Opioid Overdose Death (Under Sampling)** |  |  |  |  |  |  |  |  |
| MLR | 0.75 | 0.74 | 0.72 | 0.73 | 0.72 | 0.72 | 0.54 | 0.46 |
| LASSO | 0.76 | 0.72 | 0.72 | 0.72 | 0.72 | 0.72 | 0.51 | 0.44 |
| RF | 0.79 | 0.89 | 0.54 | 0.66 | 0.54 | 0.66 | 0.60 | 0.44 |
| GBM | 0.76 | 0.61 | 0.78 | 0.72 | 0.78 | 0.72 | 0.38 | 0.39 |
| DNN | 0.78 | 0.66 | 0.62 | 0.69 | 0.62 | 0.65 | 0.45 | 0.43 |
| **All-Cause Mortality (Unbalanced)** |  |  |  |  |  |  |  |  |
| MLR | 0.74 | 0.79 | 0.55 | 0.07 | 0.55 | 0.14 | 0.04 | 0.33 |
| LASSO | 0.74 | 0.79 | 0.55 | 0.07 | 0.55 | 0.14 | 0.04 | 0.34 |
| RF | 0.73 | 0.72 | 0.61 | 0.07 | 0.60 | 0.12 | 0.04 | 0.33 |
| GBM | 0.74 | 0.74 | 0.60 | 0.07 | 0.60 | 0.13 | 0.03 | 0.35 |
| DNN | 0.75 | 0.75 | 0.60 | 0.07 | 0.60 | 0.12 | 0.03 | 0.34 |
| **All-Cause Mortality (SMOTE)** |  |  |  |  |  |  |  |  |
| MLR | 0.59 | 0.73 | 0.43 | 0.05 | 0.43 | 0.09 | 0.09 | 0.16 |
| LASSO | 0.59 | 0.73 | 0.43 | 0.05 | 0.53 | 0.09 | 0.09 | 0.16 |
| RF | 0.69 | 0.77 | 0.50 | 0.06 | 0.50 | 0.12 | 0.15 | 0.27 |
| GBM | 0.65 | 0.76 | 0.47 | 0.06 | 0.47 | 0.10 | 0.08 | 0.24 |
| DNN | 0.70 | 0.85 | 0.41 | 0.08 | 0.41 | 0.14 | 0.07 | 0.26 |
| **All-Cause Mortality (ROSE)** |  |  |  |  |  |  |  |  |
| MLR | 0.74 | 0.71 | 0.65 | 0.07 | 0.64 | 0.12 | 0.5 | 0.36 |
| LASSO | 0.74 | 0.71 | 0.65 | 0.07 | 0.65 | 0.12 | 0.5 | 0.36 |
| RF | 0.76 | 0.73 | 0.65 | 0.07 | 0.65 | 0.13 | 0.09 | 0.38 |
| GBM | 0.74 | 0.74 | 0.62 | 0.07 | 0.62 | 0.12 | 0.42 | 0.36 |
| DNN | 0.74 | 0.67 | 0.72 | 0.07 | 0.71 | 0.12 | 0.10 | 0.38 |
| **All-Cause Mortality (Under Sampling)** |  |  |  |  |  |  |  |  |
| MLR | 0.74 | 0.61 | 0.74 | 0.64 | 0.74 | 0.69 | 0.44 | 0.35 |
| LASSO | 0.75 | 0.57 | 0.79 | 0.63 | 0.79 | 0.70 | 0.41 | 0.36 |
| RF | 0.75 | 0.74 | 0.63 | 0.69 | 0.63 | 0.66 | 0.54 | 0.37 |
| GBM | 0.76 | 0.69 | 0.71 | 0.68 | 0.71 | 0.70 | 0.51 | 0.40 |
| DNN | 0.72 | 0.70 | 0.65 | 0.67 | 0.65 | 0.66 | 0.52 | 0.34 |

^1^ AUC-ROC = Area Under the Receiver Operating Characteristic Curve; ^2^ PPV = Positive predictive value; ^3^ NPV = Negative predictive value
